# Myocardial infarction risk and tamoxifen therapy for breast cancer

**DOI:** 10.1038/sj.bjc.6602562

**Published:** 2005-04-19

**Authors:** A M Geiger, W Chen, L Bernstein

**Affiliations:** 1 Department of Research and Evaluation, Kaiser Permanente Southern California, 100 South Los Robles, 2nd Floor, Pasadena, CA 91101, USA; 2 Department of Preventive Medicine, Keck School of Medicine, University of Southern California, 1441 Eastlake Avenue, Room 4449, Los Angeles, CA 90033, USA

**Keywords:** myocardial infarction, breast cancer, tamoxifen, radiation therapy, case–control studies

## Abstract

Tamoxifen prevents recurrence after breast cancer and breast cancer among high-risk women, and may prevent myocardial infarction (MI). To assess the impact of tamoxifen on MI risk, we conducted a case–control study of first MI after breast cancer nested among women diagnosed with breast cancer, while enrolled in a health maintenance organisation from 1980 to 2000. We obtained information on breast cancer treatment and MI risk factors through medical record reviews and interviews. Data were analysed using conditional logistic regression. Of 11 045 women with breast cancer, 134 met MI criteria and were matched to two MI-free control subjects on year of birth and breast cancer diagnosis. After adjusting for smoking, hypertension and diabetes, tamoxifen was unassociated with MI (odds ratio (OR)=1.2, 95% confidence interval (CI)=0.7–1.9). Duration, cumulative dose and recency of use were not associated with MI. Radiation therapy was associated with MI (OR=2.0, 95% CI=1.1–3.5), an association that varied slightly but not statistically significantly by tamoxifen use (radiation with tamoxifen, OR=2.0, 95% CI=0.9–4.4; radiation without tamoxifen, OR=2.9, 95% CI=1.2–7.5). Tamoxifen treatment for breast cancer does not appear to increase or decrease MI risk, although radiation therapy appears to increase MI risk.

In 1978 the US Food and Drug Administration approved the nonsteroidal hormone tamoxifen for treatment of advanced postmenopausal breast cancer. Tamoxifen subsequently was shown to reduce the risk of contralateral and recurrent breast cancer, and to extend survival in women with all stages of breast cancer. (Fisher *et al*, 1998; Early Breast Cancer Trialists' Collaborative Group, 2005) In addition, tamoxifen halves the risk of breast cancer in women who are aged 35–59 years with a Gail Model (Gail *et al*, 1989) predicted 5-year breast cancer risk of 1.66% or greater, are aged 60 years and older or have lobular carcinoma *in situ* (Fisher *et al*, 1998).

Tamoxifen may have added benefit due to a cardioprotective effect. Tamoxifen appears to lower serum lipid levels in postmenopausal women with breast cancer (Love *et al*, 1994; Thangaraju *et al*, 1994) and has been associated with a pattern of inflammatory markers suggesting reduced cardiovascular disease risk (Cushman *et al*, 2001). In treatment trials, tamoxifen has been associated with reduced cardiovascular disease events, but these results have been of borderline or no statistical significance when considering only myocardial infarction (MI) (Rutqvist and Mattsson, 1993; McDonald *et al*, 1995). Tamoxifen was associated with a reduction in deaths due to MI in one trial (McDonald and Stewart, 1991), but was not statistically significantly associated with deaths due to cardiovascular disease in a systematic overview of 55 treatment trials, which enrolled 37 000 women (Early Breast Cancer Trialists' Collaborative Group, 2005). Tamoxifen appeared to have no impact on cardiovascular morbidity or mortality in two prevention trials (Reis *et al*, 2001; Cuzick *et al*, 2002). Evaluating the association of tamoxifen with MI is challenging due to the limited number of events and deaths occurring in individual trials as well as complexities in accurately identifying MI and controlling for known MI risk factors.

To assess the impact of tamoxifen treatment for breast cancer on MI risk, we conducted a case–control study nested among female Los Angeles County residents enrolled in a large health maintenance organisation when diagnosed with breast cancer. Our aims were to determine whether tamoxifen treatment for breast cancer affects MI risk, to quantify any tamoxifen effects in terms of duration, cumulative dose and recency of use, and to determine how known MI risk factors impact any tamoxifen effects.

## MATERIALS AND METHODS

### Subjects

We conducted a case–control study of MI after breast cancer nested within a cohort of female Los Angeles County residents who were diagnosed with invasive breast cancer while members of a large health maintenance organisation (Kaiser Permanente Southern California, KPSC). The study was approved by the Institutional Review Boards for the Protection of Human Subjects at KPSC and the University of Southern California, in accord with assurances filed with and approved by the US Department of Health and Human Services. Subjects provided verbal consent prior to their telephone interview.

Located at the University of Southern California Keck School of Medicine, the Los Angeles County Cancer Surveillance Program identified all women with first invasive breast cancer diagnosed at KPSC between 1 January 1980 and 1 July 2000. We linked records of these women with automated KPSC hospitalisation data from 1 January 1980 to 30 April 2001 to identify women who had possible MI after their breast cancer diagnoses. We also identified women with MI as their underlying cause of death by linking the breast cancer patient records to cancer registry follow-up data and California mortality files. Any breast cancer patient with one of the following codes as a hospitalisation discharge diagnosis or cause of death was considered a potential MI case: International Classification of Diseases 9th Revision (ICD-9) 410.x or ICD-10 I21 or I22.

Case patients were KPSC members throughout the at-risk period between their breast cancer and MI diagnoses. Patients with another cancer diagnosis (other than second primary breast cancer, cervical cancer *in situ* or basal or squamous cell skin cancer) or thromboembolic disease (stroke, venous thromboembolism or pulmonary embolism) occurring before their MI were excluded because their prior condition may have impacted their breast cancer treatment. Case patients with MI as a cause of death were assumed to have died of MI. To confirm MI identified from hospitalisation data, we used the presence or absence of chest pain, cardiac enzyme test results and electrocardiograms to classify cases as definite, probable, suspected or not acute MI following the criteria developed by the American Heart Association Council on Epidemiology (Gillum *et al*, 1984). Definite and probable MI cases were included in the study; per the criteria, these cases had either cardiac pain and abnormal enzyme test results or electrocardiograms determined to meet the criteria by the Minnesota ECG Coding Center at the University of Minnesota (http://www.epi.umn.edu/research/ecg.shtm). Myocardial infarction determinations were made blinded to the tamoxifen treatment status of the patient. Two groups of case patients were identified: women with their first incident MI after their breast cancer diagnoses and women who had MI before and after their breast cancer diagnoses.

Two control subjects were matched to each eligible case patient with their first MI after their breast cancer diagnosis. Control subjects were selected at random from breast cancer patients born within 3 years and diagnosed with breast cancer within a year as their matched case patient. In addition, control subjects had to be alive and KPSC members during their at-risk periods. Each control subject's at-risk period began with her date of breast cancer diagnosis and extended for the duration of her matched case patient's at-risk period. The end of the at-risk period defined the reference date, the time point when data collection ended for control subjects. Like case patients, a control subject was excluded if before her breast cancer or during her at-risk period she had another cancer diagnosis (other than second primary breast cancer, cervical cancer *in situ* or basal or squamous cell skin cancer). In addition, each control subject could not have pre-breast cancer evidence of thromboembolic disease (stroke, venous thromboembolism or pulmonary embolism). Any control subject with evidence of MI or other thromboembolic disease after her breast cancer diagnosis was eligible to serve as a control subject up to the day before her thromboembolic event. We confirmed the eligibility of each control subject by reviewing medical records. If a control subject became ineligible, another control was selected randomly until at least two control subjects were matched to each case patient. When fewer than two control subjects were matched to a case patient, we identified additional control subjects by first relaxing the matching criterion for year of birth to within 5 years and, if necessary, relaxed the criterion of the year of diagnosis to within 2 years. Expanded criteria were used to identify control subjects for two case patients. Despite relaxing the matching criteria, we were able to identify only one eligible control subject for each of six case patients.

### Data collection

Details of all breast cancer treatments (surgery, chemotherapy, radiation and hormonal therapy) received during the at-risk period were abstracted from medical records. In addition, we obtained information on breast tumour characteristics, oral contraceptive and hormone replacement therapy use, height and weight, and histories of hypertension, diabetes and hypercholesterolemia. Women were classified according to whether they had ever smoked or used oral contraceptives or menopausal hormone therapy, as we were unable to collect detailed information on duration of use and cumulative dose.

We attempted telephone interviews with study subjects to obtain additional information on breast cancer therapies, smoking and medical history. Next-of-kin were interviewed when subjects were deceased or unable to respond. Interviews were conducted for 96 case patients and 194 control subjects; of these interviews, next-of-kin responded on behalf of 61 case patients and 47 control subjects. Interviews could not be conducted for 38 case patients and 68 control subjects for these reasons: subject or next-of-kin refusal (23 cases, 45 controls); unable to locate subject or next-of-kin (14 cases, 14 controls); and physician denied permission to contact (one case, nine controls).

We compiled medical record and interview data into a single analytic database, using interview data only when medical record data were missing for a particular factor. We created two categories of recency of tamoxifen use at MI diagnosis (<1 year and ⩾1 year) and two categories of duration of tamoxifen use (<24 months and ⩾24 months).

### Statistical analyses

We compared case patients with first MI after breast cancer to case patients with recurrent MI after breast cancer using *χ*^2^ tests. We compared case patients with first MI after breast cancer to their individually matched control subjects using univariate and multivariate conditional logistic regression methods. Univariate and multivariate odds ratios (OR) were estimated and 95% confidence intervals (CI) were calculated (Breslow and Day, 1980). In all categorical analyses, women with missing information were included in a separate category. All multivariate analyses included categorical items for smoking (never, ever and unknown); history of hypertension (no, yes but no medication and yes received medication); history of diabetes (no, yes but no medication and yes received medication); and a combined tamoxifen and radiation therapy variable (not treated with either, received radiation therapy only, received tamoxifen only, or received both). In multivariate analyses assessing the impact of tamoxifen duration, cumulative dose and recency of use, the combined tamoxifen–radiation therapy item was replaced by items for tamoxifen duration, cumulative dose or recency of use plus an item for radiation therapy (yes/no). Similarly, in multivariate analyses assessing the impact of radiation therapy cumulative dose or side or site irradiated, the combined item was replaced by items for radiation therapy cumulative dose or side or site irradiated plus an item for tamoxifen (yes/no). All statistical tests were two-sided with a *P*-value of 0.05 considered statistically significant. All analyses were completed using SAS Version 8.02 (SAS Institute, Cary, NC, USA).

## RESULTS

**Figure 1:**
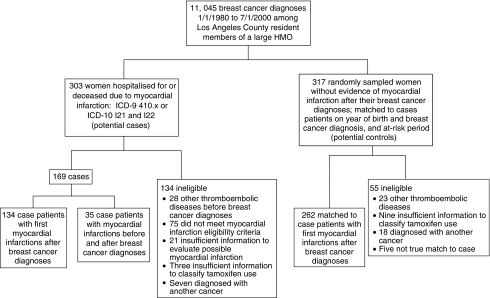
Subject eligibility determination.

A total of 11 045 female Los Angeles County residents were diagnosed with their first invasive breast cancer at KPSC between 1 January 1980 and 1 July 2000 (Figure 1). Among these women, 303 had possible MI based on hospitalisation and mortality records, with 134 who met first incident MI eligibility criteria. An additional 35 women had MI both before and after their breast cancer diagnoses. The remaining 134 women were ineligible: 28 had thromboembolic disease other than MI before their breast cancer diagnoses; 96 did not meet MI diagnostic criteria or had insufficient information for evaluation; three had insufficient tamoxifen use information; and seven had a second cancer diagnosed during their at-risk period. Of the 10 742 women without evidence of an MI after their breast cancer diagnosis, 317 were individually matched on year of birth, year of breast cancer diagnosis and at-risk period to case patient with first incident MI. We excluded 55 of these potential control subjects: 23 had thromboembolic disease other than MI before their breast cancer diagnoses; nine had insufficient tamoxifen use information; 18 had a second cancer diagnosed during their at-risk period; and five were not a true match. Thus, 262 control subjects were matched to 134 case patients.

The mean age of subjects at breast cancer diagnosis was 66.8 years (standard deviation 10.6) and mean at-risk period was 6.2 years (standard deviation 4.9); age and at-risk period distributions are shown in Table 1. Myocardial infarction occurred 1–9 years after breast cancer in 57.5% of the case patients, with 16.4% occurring within 1 year and 26.1% more than 10 years later. Over half (53.7%) of MI were classified as definite; 40 (29.9%) of case patients were identified from death certificates.

Nearly 75% of case patients and control subjects were white (Table 2). Body mass index, hypercholesterolemia and use of oral contraceptives and hormone replacement therapy were not associated with first MI after breast cancer. Myocardial infarction was associated with histories of hypertension and diabetes requiring medication (OR=2.1, 95% CI 1.3–3.4 and OR=3.0, 95% CI 1.6–5.6 respectively). Over 60% of case patients and control subjects were diagnosed with localised breast cancer; no tumour characteristic was associated with MI (Table 3).

About half of case patients and control subjects received tamoxifen therapy. In a multivariate model controlling for radiation therapy, smoking and histories of hypertension and diabetes, tamoxifen was not associated with first incident MI after breast cancer (OR=1.2, 95% CI 0.7–1.9, Table 3). Myocardial infarction was not associated with duration, cumulative dose or recency of tamoxifen use at MI (data not shown for dose and recency). Radiation therapy was associated with MI in the multivariate model (OR=2.0, 95% CI 1.1–3.5). The association of radiation therapy with MI did not vary by cumulative dose, left *vs* right side irradiated, or breast/chest *vs* other site irradiated (data not shown). When considered together in the multivariate model, the association of radiation therapy with MI was somewhat lower in tamoxifen users than in nonusers (OR=2.0, 95% CI 0.9–4.4 and OR=2.9, 95% CI 1.2–7.5, respectively) although this difference was not statistically significant (*P*=0.49). The combined effect of radiation therapy and tamoxifen on MI was consistent across levels of tamoxifen duration, cumulative dose and recency, although small sample sizes limit interpretation (data not shown for dose and recency). Chemotherapy was not associated with MI. These results did not change when stage at diagnosis was included in the model and did not vary by time between breast cancer diagnosis and MI. The association of smoking with MI was strengthened when cases identified through mortality records were removed from the analysis. There were no further changes in associations when models were restricted to case patients with definite MI (data not shown).

Case patients with MI before and after their breast cancer diagnoses were similar in age to case patients with their first MI after their breast cancer diagnoses (Table 4). Case patients with MI before and after their breast cancer diagnoses were more likely to be white and experienced their post-breast cancer MI closer in time to their breast cancer diagnoses than case patients with their first MI after their breast cancer diagnoses. Compared to case patients with first MI after their breast cancer diagnoses, case patients with MI before and after their breast cancer diagnoses were more likely to have been treated for hypertension and diabetes and to have ever smoked. Use of adjuvant tamoxifen and radiation therapy was similar in the two case patient groups.

## DISCUSSION

We found that adjuvant tamoxifen therapy was not associated with first MI after breast cancer, regardless of duration, cumulative dose or recency of use. Myocardial infarction was associated with radiation therapy and was more common in smokers and women with histories of medication-treated hypertension or diabetes. We did not detect any association between MI and radiation therapy cumulative dose or side or site irradiated. The association of radiation therapy with MI appeared reduced slightly among tamoxifen users, but we did not have sufficient statistical power to demonstrate that the effect differed from nonusers. These results inform clinical decision-making and are pertinent to weighing the relative risks and benefits of tamoxifen *vs* other treatments like aromatase inhibitors.

Despite evidence that tamoxifen lowers serum lipid levels in postmenopausal women with breast cancer (Love *et al*, 1994; Thangaraju *et al*, 1994) and is associated with a pattern of inflammatory markers suggesting reduced cardiovascular disease risk (Cushman *et al*, 2001), tamoxifen trials have not demonstrated a consistent, statistically significant reduction in morbidity or mortality due to MI. McDonald *et al* (1995) reported a hazard ratio of 1.92 (95% CI 0.99–3.73) for MI in women randomised to the control arm relative to those randomised to the tamoxifen arm. Rutqvist and Mattsson (1993) reported a relative hazard of 0.83 (95% CI 0.45–1.56) for MI in tamoxifen arm patients relative to control arm patients, and noted no differences by duration of tamoxifen therapy. One treatment trial reported an association between tamoxifen and reduced MI deaths (McDonald and Stewart, 1991), but a systematic overview of 55 randomised trials (Early Breast Cancer Trialists' Collaborative Group, 2005) failed to confirm this association. Prevention trials have not reported an association between tamoxifen and cardiovascular disease (Reis *et al*, 2001; Cuzick *et al*, 2002). Using stringent MI diagnostic criteria and controlling for known MI risk factors, our results provide no support for an association between tamoxifen use and first MI after breast cancer.

Studies examining a possible association of radiation therapy with MI have conflicting results. An overview of early breast cancer radiation therapy trials reported an association with vascular mortality in breast cancer survivors (Early Breast Cancer Trialists' Collaborative Group, 2000). Rutqvist and Johansson (1990) reported that radiation therapy for left-sided breast cancer given in 1970–1980 resulted in a statistically significant higher mortality due to MI. Paszat *et al* (1998) reported an association of radiation therapy with fatal MI in women with left-sided cancer diagnosed from 1973–1992, particularly among women under age 60 years. Yet two studies of women treated in the 1980s reported no association of radiation therapy with MI morbidity or mortality (Hojris *et al*, 1999; Vallis *et al*, 2002). Study differences in length of patient follow-up, changes in radiation therapy techniques over time and a lack of control for other risk factors may explain the variable findings. Right-sided radiation exposures may contribute to increased MI risk due to scatter, or this may be a spurious finding. The results of our study using confirmed MI and controlling for other risk factors support the possibility that radiation therapy is associated with increased MI risk, but cannot confirm this association due to the majority of our patients receiving mastectomy, leaving small numbers of patients eligible for radiation therapy. In addition, our study was not designed to examine associations between radiation therapy and MI, and thus did not include an effort to estimate radiation dose to the heart; future studies should include this approach.

For the majority of our subjects we gathered detailed information on tamoxifen use from medical records, information not impacted by self-report bias. Nevertheless, we were unable to accurately classify tamoxifen use for a small proportion of study subjects due to insufficient medical record information. In addition, small numbers limit our ability to reach conclusions about the relative effects of short- and long-course tamoxifen use.

While randomised controlled trials are the strongest design for determining treatment efficacy, the application of trial results may be limited when trial conditions differ from clinical practice (Fletcher, 2002). In this common situation, observational studies provide useful information for application of trial results to clinical practice (Vijan *et al*, 2000). The interpretation and application of tamoxifen treatment trial results are hampered by volunteer bias and stringent eligibility criteria that create systematic differences between trial participants and the population of all women with breast cancer. In addition most tamoxifen trials have limited ability to collect data on anything other than primary end points, thus secondary diagnoses are not confirmed and information on risk factors is unavailable. Owing to potential selection bias and misclassification of exposure and outcome variables, trial-based estimates of MI incidence after breast cancer treatment are likely inaccurate. We conducted a population-based study in which we were able to include women without regard to willingness to participate in a trial and without any exclusion criteria, thus enhancing the generalisability of our results to clinical practice. In addition, by conducting our study within an integrated health care system, we were able to identify and confirm MI diagnoses systematically as well as gather detailed information on known MI risk factors. We likely identified all MI because KPSC has a safe and effective system for returning members hospitalised at non-KPSC facilities to KPSC facilities (Selevan *et al*, 1999). We approximated randomisation by matching on age, year of diagnosis and at-risk period.

To date, randomised trials have reported mixed results on the association of MI morbidity and mortality with adjuvant tamoxifen use. In our case–control study of MI after first invasive breast cancer diagnosis nested among female residents of Los Angeles County enrolled in a large health maintenance organisation, we found no association of adjuvant tamoxifen use with first MI, including no impact of duration, cumulative dose or recency of use. Our results complement trial results by documenting that among a general population of women diagnosed with breast cancer, tamoxifen treatment does not appear to increase or decrease MI risk, and suggest that prevention of MI should not be considered an advantage of tamoxifen use. In addition, we identified an association of radiation therapy and MI, an association reported in some but not all prior studies. Consistent with Constine and Lipshultz (2004), we believe that women with a history of radiation therapy or any other potentially cardiotoxic therapy for breast cancer will benefit from ongoing risk factor monitoring and lifestyle modifications to improve cardiovascular health.

## Figures and Tables

**Table 1:** Subject characteristics

	**Case patients with first MI after breast cancer (*n*=134)**	**Controls without MI (*n*=262)**
**Characteristic**	***N*** **(%)**	***N*** **(%)**
*Age at breast cancer diagnosis (years)*		
<50	10 (7.5)	16 (6.1)
50–59	23 (17.2)	48 (18.3)
60–69	46 (34.3)	91 (34.7)
70–79	42 (31.3)	86 (32.8)
⩾80	13 (9.7)	21 (8.0)
		
*Year of breast cancer diagnosis*		
1980–1984	40 (29.9)	75 (28.6)
1985–1989	49 (36.6)	96 (36.6)
1990–1994	27 (20.2)	59 (22.5)
1995–1998	18 (13.4)	32 (12.2)
		
*Years between initial breast cancer and MI diagnoses* a		
<1	22 (16.4)	42 (16.0)
1–3	34 (25.4)	69 (26.3)
4–6	26 (19.4)	52 (19.9)
7–9	17 (12.7)	32 (12.2)
⩾10	35 (26.1)	67 (25.6)
		
*MI classification*		
Definite	72 (53.7)	Not applicable
Probable	22 (16.4)	
Immediate fatality^b^	40 (29.9)	

a As control subjects had no MI, data are from the reference date that marks the end of control subjects' at-risk period, which began at control subjects' dates of breast cancer diagnosis and extended for the duration of the matched case patients' time from breast cancer to MI diagnoses.

b Cases identified from death certificate only; no diagnostic work-up to permit classification.

**Table 2:** Odds ratios (OR) and 95% confidence intervals (95% CI) of first myocardial infarction (MI) after breast cancer diagnosis associated with subject characteristics and medical history

**Characteristics**	**# of cases/# of controls**	**Univariate OR (95% CI)**	**Multivariate^a^ OR (95% CI)**
*Race/ethnicity*			
Black	19/45	0.8 (0.5–1.5)	0.6 (0.3–1.1)
White	97/191	1.0 (referent)	1.0 (referent)
Other/unknown	18/26	1.4 (0.7–2.6)	1.2 (0.6–2.5)
			
*Body mass index at MI (kg/m^2^)* b			
<25	52/105	1.0 (referent)	1.0 (referent)
25–29.9	38/79	0.9 (0.6–1.6)	0.8 (0.4–1.4)
>30	31/58	1.1 (0.6–1.9)	0.8 (0.4–1.5)
Unknown	13/20		
			
*Oral contraceptive use ever*			
No	93/201	1.0 (referent)	1.0 (referent)
Yes	25/43	1.5 (0.8–3.0)	1.5 (0.7–3.0)
Unknown	16/18		
			
*Use of oestrogen and/or hormone replacement therapy ever*			
No	60/105	1.0 (referent)	1.0 (referent)
Yes	69/147	0.8 (0.5–1.2)	0.9 (0.5–1.4)
Unknown	5/10		
			
*Smoking status at MI* b			
Never	70/156	1.0 (referent)	1.0 (referent)
Ever	59/100	1.3 (0.8–2.0)	1.5 (0.9–2.4)
Unknown	5/6		
			
*History of hypertension*			
No	34/111	1.0 (referent)	1.0 (referent)
Yes, no medication	7/28	0.8 (0.3–2.1)	0.7 (0.3–2.0)
Yes, medication	93/123	2.5 (1.5–4.0)	2.1 (1.3–3.4)
			
*History of diabetes*			
No	88/217	1.0 (referent)	1.0 (referent)
Yes, no medication	8/13	1.5 (0.6–4.2)	1.5 (0.6–4.2)
Yes, medication	38/32	3.2 (1.8–5.6)	3.0 (1.6–5.6)
			
*History of hypercholesterolemia*			
No	68/150	1.0 (referent)	1.0 (referent)
Yes, no medication	34/76	1.0 (0.6–1.8)	1.1 (0.6–2.0)
Yes, medication	32/36	2.0 (1.1–3.7)	1.4 (0.7–2.7)

a Multivariate model includes categorical items for the named characteristic, histories of hypertension and diabetes, smoking and a combined item for tamoxifen and radiation treatment.

b As control subjects had no MI, data are from the reference date that marks the end of control subjects' at-risk period, which began at control subjects' dates of breast cancer diagnosis and extended for the duration of the matched case patients' time from breast cancer to MI diagnoses.

**Table 3:** Odds ratios (OR) and 95% confidence intervals (95% CI) of first myocardial infarction after breast cancer diagnosis associated with tumor and treatment characteristics

**Characteristics**	**# of cases/# of controls**	**Univariate OR (95% CI)**	**Multivariate^a^ OR (95% CI)**
*Stage at diagnosis*			
Localised	89/183	1.0 (referent)	1.0 (referent)
Regional	38/73	1.1 (0.7–1.7)	1.0 (0.6–1.8)
Distant/unstaged	7/6	2.5 (0.8–8.0)	2.2 (0.6–7.7)
			
*Oestrogen receptor status*			
Negative	20/45	1.0 (referent)	1.0 (referent)
Positive or borderline	86/154	1.3 (0.7–2.4)	1.3 (0.6–2.6)
Not done	28/63		
			
*Progesterone receptor status*			
Negative	33/73	1.0 (referent)	1.0 (referent)
Positive or borderline	61/110	1.3 (0.8–2.1)	1.1 (0.6–1.9)
Not done	40/79		
			
*Lymph nodes involved*			
No	75/155	1.0 (referent)	1.0 (referent)
Yes	37/71	1.1 (0.7–1.7)	1.0 (0.6–1.9)
No nodal surgery	22/36		
			
*Tamoxifen therapy ever* b			
No	65/132	1.0 (referent)	1.0 (referent)
Yes	69/130	1.1 (0.7–1.8)	1.2 (0.7–1.9)
			
Duration of use (months)			
None	65/132	1.0 (referent)	1.0 (referent)
<24	29/47	1.5 (0.8–2.9)	1.6 (0.8–3.2)
⩾24	35/79	0.9 (0.5–1.5)	0.9 (0.5–1.7)
Unknown	5/4		
			
*Radiation therapy* b			
No	101/223	1.0 (referent)	1.0 (referent)
Yes	33/39	1.8 (1.1–3.1)	2.0 (1.1–3.5)
			
Radiation cumulative dose (rads)^c^			
None	101/223	1.0 (referent)	1.0 (referent)
⩽5000	18/18	2.1 (1.04–4.4)	2.2 (1.01–4.8)
>5000	14/20	1.5 (0.7–3.1)	1.7 (0.7–3.8)
			
Radiation side			
None	101/223	1.0 (referent)	1.0 (referent)
Right	13/14	2.0 (0.9–4.8)	2.1 (0.8–5.3)
Left	20/25	1.7 (0.9–3.2)	1.9 (0.9–3.9)
			
*Tamoxifen and radiation therapy*			
Neither	52/120	1.0 (referent)	1.0 (referent)
Radiation therapy only	13/12	2.5 (1.02–5.9)	2.9 (1.2–7.5)
Tamoxifen only	49/103	1.2 (0.7–2.0)	1.3 (0.8–2.3)
Both	20/27	1.8 (0.9–3.6)	2.0 (0.9–4.4)
			
*Tamoxifen months of use and radiation therapy* b			
Neither	52/120	1.0 (referent)	1.0 (referent)
Radiation therapy only	13/12	2.5 (1.04–6.1)	2.9 (1.1–7.5)
No radiation therapy			
Tamoxifen			
<24	18/36	1.5 (0.7–3.0)	1.7 (0.8–3.7)
⩾24	26/64	1.0 (0.5–1.8)	1.1 (0.6–2.1)
Radiation therapy			
Tamoxifen			
<24	11/11	2.4 (0.9–6.4)	3.2 (1.1–9.1)
⩾24	9/15	1.4 (0.5–3.7)	1.4 (0.5–4.0)
Tamoxifen months unknown	5/4		
			
*Chemotherapy*			
No	109/218	1.0 (referent)	1.0 (referent)
Yes	25/44	1.2 (0.6–2.1)	1.2 (0.6–2.3)

a All multivariate models include categorical items for histories of hypertension and diabetes, smoking and a combined item for tamoxifen and radiation treatment.

b In multivariate model, the combined tamoxifen-radiation therapy item was replaced by separate items. For example, the model to examine tamoxifen duration of use included the radiation therapy (yes/no) item.

c Subjects with details unknown removed (one case with matched controls, one control).

**Table 4:** Comparison of case patients with first myocardial infarction (MI) after breast cancer *vs* case patients with MI before and after breast cancer

	**Case patients with first MI after breast cancer (*n*=134)**	**Cases with MI before and after breast cancer (*n*=35)**	
**Characteristic**	***N*** **(%)**	***N*** **(%)**	***P***
*Year of breast cancer diagnosis*			0.18
1980–1984	40 (29.9)	5 (14.3)	
1985–1989	49 (36.6)	12 (34.3)	
1990–1994	27 (20.2)	11 (31.4)	
1995–1998	18 (13.4)	7 (20.0)	
			
*Years between initial breast cancer and post-breast cancer MI diagnosis*			0.011
<1	22 (16.4)	11 (31.4)	
1–3	34 (25.4)	11 (31.4)	
4–6	26 (19.4)	7 (20.0)	
7–9	17 (12.7)	6 (17.1)	
⩾10	35 (26.1)	0 (0.0)	
			
*MI classification*			0.62
Definite	72 (53.7)	18 (51.4)	
Probable	22 (16.4)	4 (11.4)	
Immediate fatality^a^	40 (29.9)	13 (37.1)	
			
*Age at breast cancer diagnosis (years)*			0.13
<50	10 (7.5)	1 (2.9)	
50–59	23 (17.2)	1 (2.9)	
60–69	46 (34.3)	13 (37.1)	
70–79	42 (31.3)	14 (40.0)	
>80	13 (9.7)	6 (17.1)	
			
*Race/ethnicity*			0.072
Black	19 (14.2)	6 (17.1)	
White	97 (72.4)	29 (82.9)	
Other/unknown	18 (13.4)	0 (0.0)	
			
*Oral contraceptive use ever*			0.12
No	93 (69.4)	21 (60.0)	
Yes	25 (18.7)	5 (14.3)	
Unknown	16 (11.9)	9 (25.7)	
			
*Use of oestrogen and/or hormone replacement therapy ever*			0.83
No	60 (44.8)	14 (40.0)	
Yes	69 (51.5)	20 (57.1)	
Unknown	5 (3.7)	1 (2.9)	
			
*Smoking status at MI*			0.004
Never	70 (52.2)	9 (25.7)	
Ever	59 (44.0)	21 (60.0)	
Unknown	5 (3.7)	5 (14.3)	
			
*History of hypertension*			0.091
No	34 (25.4)	3 (8.6)	
Yes, no medication	7 (5.2)	3 (8.6)	
Yes, medication	93 (69.4)	29 (82.9)	
			
*History of diabetes*			0.18
No	88 (65.7)	1 (48.6)	
Yes, no medication	8 (6.0)	3 (8.6)	
Yes, medication	38 (28.4)	15 (42.9)	
			
*History of hypocholesterolemia*			0.82
No	68 (50.8)	16 (45.7)	
Yes, no medication	34 (25.4)	9 (25.7)	
Yes, medication	32 (23.9)	10 (28.6)	
			
*Stage at diagnosis*			0.99
Localised	89 (66.4)	23 (65.7)	
Regional	38 (28.4)	10 (28.6)	
Distant/unstageable	7 (5.2)	2 (5.7)	
			
*Oestrogen receptor status*			0.26
Negative	20 (14.9)	2 (5.7)	
Positive or borderline	86 (64.2)	27 (77.1)	
Not done	28 (20.9)	6 (17.1)	
			
*Progesterone receptor status*			0.17
Negative	33 (24.6)	5 (14.3)	
Positive or borderline	61 (45.5)	22 (62.9)	
Not done	40 (29.9)	8 (22.9)	
			
*Tamoxifen and radiation therapy*			0.15
Neither	52 (38.8)	8 (22.9)	
Radiation therapy only	13 (9.7)	2 (5.7)	
Tamoxifen only	49 (36.6)	20 (57.1)	
Both	20 (14.9)	5 (14.3)	

a Cases identified from death certificate only; no diagnostic work-up to permit classification.
